# Seeded Bayesian Networks: Constructing genetic networks from microarray data

**DOI:** 10.1186/1752-0509-2-57

**Published:** 2008-07-04

**Authors:** Amira Djebbari, John Quackenbush

**Affiliations:** 1 Department of Biostatistics and Computational Biology, Dana-Farber Cancer Institute and Department of Biostatistics, Harvard School of Public Health, Boston, MA 02115, USA

## Abstract

**Background:**

DNA microarrays and other genomics-inspired technologies provide large datasets that often include hidden patterns of correlation between genes reflecting the complex processes that underlie cellular metabolism and physiology. The challenge in analyzing large-scale expression data has been to extract biologically meaningful inferences regarding these processes – often represented as networks – in an environment where the datasets are often imperfect and biological noise can obscure the actual signal. Although many techniques have been developed in an attempt to address these issues, to date their ability to extract meaningful and predictive network relationships has been limited. Here we describe a method that draws on prior information about gene-gene interactions to infer biologically relevant pathways from microarray data. Our approach consists of using preliminary networks derived from the literature and/or protein-protein interaction data as seeds for a Bayesian network analysis of microarray results.

**Results:**

Through a bootstrap analysis of gene expression data derived from a number of leukemia studies, we demonstrate that seeded Bayesian Networks have the ability to identify high-confidence gene-gene interactions which can then be validated by comparison to other sources of pathway data.

**Conclusion:**

The use of network seeds greatly improves the ability of Bayesian Network analysis to learn gene interaction networks from gene expression data. We demonstrate that the use of seeds derived from the biomedical literature or high-throughput protein-protein interaction data, or the combination, provides improvement over a standard Bayesian Network analysis, allowing networks involving dynamic processes to be deduced from the static snapshots of biological systems that represent the most common source of microarray data. Software implementing these methods has been included in the widely used TM4 microarray analysis package.

## Background

DNA microarrays and other genome-inspired, high-throughput technologies allow us to capture information regarding gene expression across the entire collection of genes in an organism. While it has long been argued that such genome-wide profiles should allow the identification of networks and pathways, deducing such interactions for even a small number of genes remains a daunting task. Although several gene network modeling techniques have been applied to microarray data, including weight matrices [1], Boolean networks [2], and differential equations [3], Bayesian Networks (BNs), BN seemed to show the greatest promise in the analysis of expression data as they provided the ability to learn network structures and develop predictive models of system response [4].

**Figure 1:**
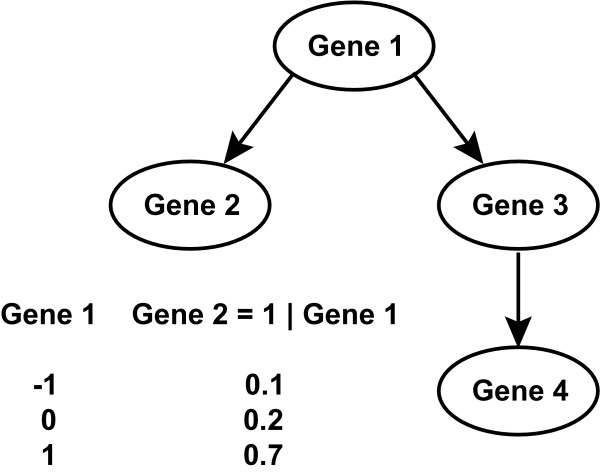
**A Bayesian network example where each random variable corresponds to a gene that can take one of three states corresponding to its transcriptional response: -1 for under-expressed, 0 for unchanged, and +1 for over-expressed.** The table represents a subset of the complete set of conditional probabilities for Gene2, here indicating the likelihood that Gene2 is up-regulated given the transcriptional state of Gene1.

In the BN formalism, a network of interacting genes is represented as a graph in which the genes are "nodes" and interactions between genes are "edges"; the terms network and graph are often used interchangeably and in a BN framework, the edges are directed. As an example, consider a simple graph in which a node, Gene1, is the only parent of a second node, Gene2 (Figure 1). Associated with the edge between them is a conditional probability table that provides estimates of the likelihood of the state of Gene2 given the state of Gene1. For instance, the probability of Gene2 being over-expressed given that Gene1 is over-expressed is 0.7, which may be interpreted as implying Gene1 activates Gene2. Placing this into a formal context, a Bayesian Network is defined to be a pair *(G, θ) *where *G *is a directed acyclic graph (DAG) whose vertices are random variables *X*_1_,...,*X*_*n *_and *θ *is the conditional distribution for each variable given its parents *P(X*_*i *_|*Parents(X*_*i*_*))*. Bayesian networks only allow dependencies between a node and its parents and conditional independence statements encoded by the network structure define the conditional probability distributions; in the case of genes, the factors that influence its expression.

BNs were first applied to gene expression studies in the analysis of the yeast cell cycle [4], a dataset that consisted of expression data collected over a carefully planned time-course [5]. Friedman and colleagues were able to deduce a predictive model of the cell cycle machinery in yeast from this data, a result that generated a great deal of excitement within the research community. However, application of BN analysis to more "realistic" datasets (i.e. tumor vs. normal, treated vs. control) failed to provide similarly useful insight and as such is rarely applied in analysis of expression profiling data.

Application of Bayesian Network analysis in genomics is challenging for a number of reasons. The first problem is that learning BNs is computationally expensive as, ideally, one must assess all potential network topologies corresponding to all possible sets of directed acyclic graphs linking the genes. This results in a combinatorial explosion of the number of possible structures and parameters; formally this has been shown to be an NP-Complete problem [6]. As an alternative, general purpose heuristic search algorithms, such as greedy hill climbing, can be used to explore the "state space" of the problem (here, the relative expression state for each gene in each sample) in an attempt to optimize some scoring function. The problem with these approaches is that they often find local maxima and do not converge to the globally optimal solution. This accounts, in most instances, for the failure of BN analysis to find "realistic" networks in datasets that lacked the richness of the cell cycle time course.

A potential solution to the limitations of the type of general-purpose search algorithms was proposed by Wolpert and Macready, who noted that the use of domain-specific knowledge can provide a useful bias that can lead to near-optimal solutions in exploring the state space of a particular problem [7]. In the context of BN analysis of microarray data, a useful bias can be introduced through the use of preliminary network topologies as soft constraints to seed the search for a network graph [8-10], an approach that has been applied in a variety of related problems [11-15]. Although a network seed biases the search for the best topology, it does not limit it so that new potential interactions between genes can be identified.

A number of possibilities exist to provide prior seeds for the network topologies, including pathway/interaction databases, networks deduced from the published biomedical literature indexed in PubMed, and those constructed from high-throughput interaction screens such as the protein-protein interaction (PPI) described by Rual and colleagues [16]. As our goal, in part, is to discover new interactions, we chose to concentrate on prior networks deduced from the literature and PPI data which often include potential interactions not yet annotated in curated pathway databases. As such, this approach has potential to discover new interactions by combining diverse sources of data and information.

A second challenge in the application of methods such as BN analysis to microarrays is the typical design of genomics studies. As noted previously, most microarray studies do not involve uniform temporal sampling of the state of a system where inferred relationships in the gene expression state space of genes can more easily be detected. Rather, typical studies involve static comparisons of different phenotypic or treatment groups where relationships between gene states can be much more subtle. Further, and possibly more importantly, the large number of genes assayed on a single array and the relatively small number of samples profiled generally provide too few measurements to constrain potential models, and arrive at an optimal solution. To address this, we implement model averaging through bootstrapping which allows us to compute confidence estimates for network features in the models we derive.

Ultimately, the question we must resolve is whether application of BN analysis to gene expression data can reveal useful networks that can lead to testable hypotheses about the state and response of the systems under study. In particular, our goal in this manuscript is to assess the use of prior network structures as seeds for a search of the gene expression state space. To do this, we present an analysis of two leukemia datasets [17-19]. As described below, we find that by combining microarray data with prior network structures deduced from the literature, PPI, or a combination of these, we can better recover known pathways and relationships between genes than when analyzing microarray data without a network seed. To estimate the robustness of our approach, we compare the learned networks to known networks from the KEGG database [20] and show that networks constructed with high confidence edges have a very small false-positive rate, but at the expense of missing true edges. This suggests that even when applied to imperfect data, our approach provides a conservative way of recovering pathways and of identifying potential new interactions that can be further evaluated in the laboratory.

## Results

Our analytical pipeline closely follows that outlined by Friedman and colleagues [4], with important modifications. One starts with normalized DNA microarray data from an appropriate study and identifies a set of candidate genes that will be further analyzed. For example, one might use a variety of statistical methods to identify genes correlating with the various phenotypic states under study or select a set of genes representing a particular pathway or process thought relevant to the system being analyzed. The expression data for these genes are then discretized using a multinomial model [4] and assigned to three mutually exclusive and exhaustive bins (under-expressed, unchanged, and over-expressed) by equal-width binning. This discretized data then provides the "raw material" that is subsequently used to learn a Bayesian Network. Our modifications of the Friedman *et al*. approach involve the introduction of a prior network seed to initiate the learning stage and the use of bootstrapping to estimate confidence in the equivalence classes of final networks. As a means of comparing a BN analysis both with and without network seeds, we compared the resulting networks to "known" pathways and evaluated our ability to reproduce documented interactions between genes.

### Deducing prior network structure from the published literature

The published biomedical literature represents nearly the entirety of our existing knowledge of biological entities and the relationships between them. There are a variety of methods that can be used to infer relationships between genes based on published results. For the purpose of the analysis presented here, we chose a simple but effective way of inferring potential functional associations between genes, the co-occurrences method described by Jensen and colleagues [21]. Quite simply, if two and only two genes are described in a single article indexed in PubMed [22], then one assumes an interaction; weights are assigned to interactions based on the relative number of articles mentioning those genes together. Formally, we create a graph in which nodes representing genes are connected by an edge. For two genes, *A *and *B*, we assign a co-occurrence edge weight, which counts the number of times an "interaction" appears in the literature relative to the total number of manuscripts surveyed, as prior probabilities:

$$p(A,B)=\frac{w(A,B)}{\underset{e\in E}{\max}w(e)}$$

where *w(A, B) *and *w(e) *denote the weight of edge *(A, B) *and the weight of edge *e *respectively in the set of edges E.

The choice of limiting edge assignments to genes appearing pair-wise in articles indexed in PubMed was based on an analysis of the network properties that can be deduced from the literature. Specifically, limiting networks to papers containing two and only two genes results in network topologies exhibiting a scale-free behavior. In contrast, increasing the threshold beyond two genes introduced deviations from scale-free behavior, leading to networks that have properties of a complete graph (results not shown). Further, setting some threshold is necessary to remove publications including whole-genome studies that mention thousands of genes and consequently produce networks where each node is connected to every other node. The choice of two, while conservative, allows for a prior network with the highest possible confidence without resorting to more ambitious text-mining approaches.

### Deducing prior network structure from high-throughput screens

There are many sources of network priors other than the literature, including the growing collection of interactome data available from high-throughput yeast two hybrid protein-protein interaction (PPI) screens such as that recently released by Rual and colleagues [16]. While these datasets are still sparse – current interactome datasets are thought to represent only about 1% of the human interactome (2754 edges from approximately 64 million protein pairs) – they represent an unbiased screen for interactions and have identified many not catalogued in the published literature. Since we were interested in exploring the usefulness of PPI-based networks as priors, we chose to compensate for their relative sparseness by using the "significant gene" set derived from our initial microarray analysis as a starting point to search the PPI data and allowed the initial networks to expand through protein interactions at a distance at most *k *= 3 interactions away using Floyd's all-pairs shortest paths algorithm [23]. For PPI networks, we use a uniform distribution for the prior probabilities for all edges.

### Setting an initial directionality for network edges

As a BN is a directed acyclic graph (DAG), the initial network used to seed the search must have directions assigned to each edge. To address this for the undirected literature and PPI networks, we developed an approach based on a modified depth-first search (DFS) algorithm to traverse the graph and make assignments. Simply, one starts at some randomly selected node and explores the graph as far as possible along each branch before backtracking, assigning directions as the graph is traversed. Because DFS is commonly used for cycle detection [23], we modified the standard algorithm to keep edges in the depth-first tree but direct back edges in increasing order of visiting timestamp to ensure the resulting graph is a DAG (the modified DFS, along with an alternate approach, is described in detail in Supplement S1; see Additional File 1). While all of the resulting edges may not be directed appropriately, this is not a problem as the BN search considers not only additions and deletions of edges but also edge reversals and therefore provides a means of correcting an error made at this stage. As this method cannot estimate the conditional probabilities on the edges, we assume a flat distribution such that all interactions are, initially, equally likely.

### Bootstrapping

As noted previously, overfitting is a potential problem in BN analysis, particularly given the small number of samples profiled in a typical microarray experiment. To compensate for this, we perform model averaging through non-parametric bootstrapping (resampling with replacement) to estimate the confidence in various network features learned [24]. As this requires comparison of the DAGs derived from each bootstrap iteration, one must consider the equivalence class of DAGs. An equivalence class is a partition of a set into subsets that are equivalent under some operation, such as the partition of the integers into evens and odds under the modulo-two operation. Here we encode the independencies within the DAGs and use these as way of partitioning the entire collection into subsets in which each member is equivalent. As described in greater detail in the Supplement S2 (see Additional File 1), we transform the DAG from each bootstrap iteration into a complete partially directed acyclic graph (CPDAG) which is a representative of an equivalence class of DAGs through the DAG-to-CPDAG algorithm described by Chickering [25]. For each CPDAG, we check whether the features of interest [directed edge, undirected edge, order relation (one variable is the ancestor of the other variable), and Markov relation (if two variables are connected either way or if they are both parents of another variable)] are found. By counting how often a particular feature occurs relative to the total number of iterations, we can estimate the overall bootstrap confidence and consequently select features that are strongly supported by the data.

In the work presented here, we perform 100 bootstrap operations for each of four cases: gene expression data alone (with no priors), or using priors derived from the literature, from PPI data, or from a combination of the two. For each iteration, we use the open-source WEKA package [26,27] with a greedy hill climbing algorithm to optimize the BDe score [28], a Bayesian metric that uses an explicit prior over networks and is proportional to the posterior probability of the network given the data; as a default, we set the maximum number of parents for each node equal to three. In this framework, both the network topology and the conditional probabilities associated with each edge are learned from the data, but starting from our initial seed network.

To evaluate the performance of this approach, we investigate a number of features that can be learned from the data and estimate the confidence, as noted, based on the frequency with which it occurs. We use this to address three questions: Can we recover known gene interactions? Are the confidence estimates different in prior (combined literature and microarray data) versus no prior (microarray data alone)? Is the performance better when using prior rather than the no prior approach?

Finally, it should be noted that there are a number of alternative scoring metrics and search algorithms. The choice here in part reflects the initial presentation by Friedman [4] but also represents the defaults defined by WEKA. We investigated a number of alternatives and each produced a result consistent with that described below; an overall improvement in the resulting networks when using an initial seed network structure.

### BN analysis of gene expression in Leukemia I: an example

As a first test of seeded BN approach, we analyzed a well-known example of a typical microarray expression study, the 1999, Golub *et al*. comparison of Acute Lymphoblastic Leukemia (ALL) with that in Acute Myeloid Leukemia (AML) patients (38 samples, 27 ALL and 11 AML) that used the Affymetrix Hu6800 GeneChip™ (containing 7129 gene-specific probe sets representing approximately 6817 genes) to identify signatures that could differentiate these disease subtypes [17]. As is the case in most analyses of this sort, we first performed a statistical filtering to reduce the complexity of the data. Here we simply used a between-subjects t-test with a standard Bonferroni correction to identify the 40 genes most significant for distinguishing the ALL and AML samples. As noted previously, we expanded the seed networks to take better advantage of the limited PPI data by including those genes a distance of three or fewer from the initial set (based on the PPI data). This PPI-extended dataset consisted of 63 genes and served as the starting point for analysis (See Supplement S3; see Additional File 1); the resulting literature plus PPI "seed" network, included 44 gene nodes connected by 48 edges and 19 singleton (unconnected) genes.

**Figure 2:**
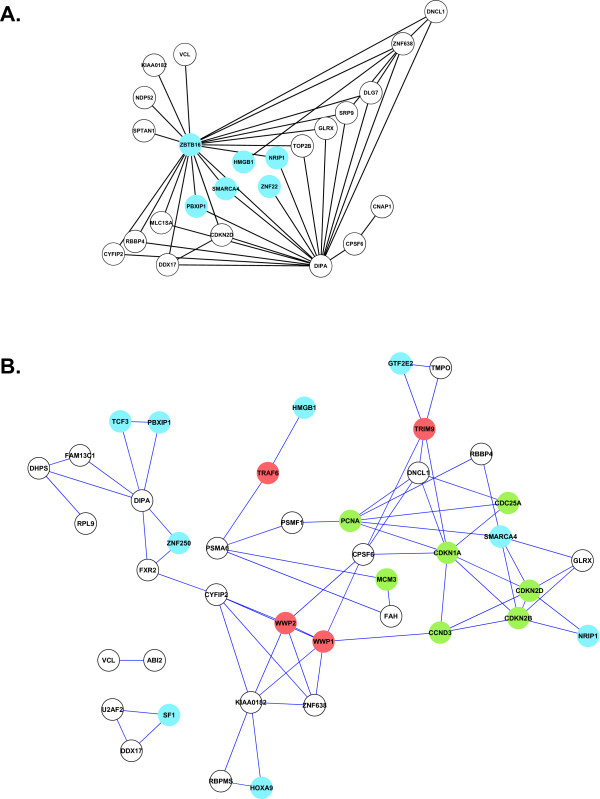
**Networks arising from a Bayesian Network analysis of gene expression data of Golub *et al*. **[17]** and rendered in Cytoscape **[29]** using (A) no prior information and (B) prior network seeds deduced from a combination of the literature and the protein-protein interaction data of Rual *et al*. **[16]. In both cases, the BNs were learned using a greedy hill climbing algorithm to optimize the BDe score. Shown here are edges representing the Markov relation between genes with confidence scores of at least 0.70 after 100 bootstrap iterations. In (A), genes highlighted in blue are involved in regulation of transcription; no other clear functional class is represented. This network is comprised of 24 nodes, 41 edges; relative to the network one could postulate based on the literature and PPI data it is missing 42 edges and contains 41 "extra" edges. For (B), genes highlighted in blue are involved in regulation of transcription, those in green are involved in cell cycle, and genes in red are involved in ubiquitination. Compared with the literature and PPI network used as a prior, this network, containing 41 nodes and 68 edges, has 0 missing edges, and 25 extra edges.

Using the approach described above, we ran 100 bootstrap iterations and collected features with bootstrap confidence greater that 0.7 (occurring in more than 70% of iterations); the resulting networks were visualized using Cytoscape [29,30]. Figure 2A shows the results of a BN analysis for the 63 starting genes without the use of a seed prior, while Figure 2B shows the resulting network when a prior seed based on both the literature and PPI data is used. What is surprising is that when using both the literature and PPI as priors, genes involved in cell cycle are found at the core of the network whereas genes involved in regulation of transcription and ubiquitination, which is known to be involved in protein degradation, are on the periphery. Furthermore, most of these gene interactions are directly or indirectly involved in Rb/E2F pathway [31,32], which is reflected in observed cell cycle differences between ALL and AML [33].

It is also instructive to examine quantitative measures such as the BDe score to evaluate BN performance. Table 1 shows the BDe scores for the networks derived from microarray data alone as well as when using prior network seeds derived from the literature, PPI data, and a combination of the two. While, in general, higher scoring networks are more likely given the data, the relatively small difference in scores makes it difficult to use this as an objective performance metric. Indeed, the greedy hill climbing approach is known to have potential to overfit the data, particularly given limited data, and such non-specialized optimization approaches are known to often provide non-useful networks [34].

**Table 1:** Scores for learned networks using top 40 genes and those at distance at most 3 from them in PPI using the leukemia dataset from Golub *et al*. [17] using microarray data only or priors from combinations of the literature and PPI data.

Prior Seed Source	None	Literature prior	PPI prior	Literature and PPI prior
Score	-29.1340899	-29.82373161	-29.90025881	-30.99743368

When using the priors however, we believe that network confidence is a better measure of overall performance. Tables 2 and 3 show the average confidence for the directed edge and Markov relation features, respectively, for the seven gene pairs with the highest confidence. Clearly the use of prior network seeds greatly increases our ability to recover known interactions. This is further illustrated below.

**Table 2:** Average confidence values for directed edge features for the 7 highest-confidence gene pairs derived from the Golub *et al*. data [17] in a BN analysis using no prior seeds or priors derived from the literature. The t-test p-value measures the likelihood that prior and no-prior results are equivalent.

*Gene pairs*		*Average no prior*	*Average prior*	*t-test p-value*
			
SMARCA4	SMARCA4_2	0.4075	0.959583	0.001059
CBX1	SRP9	0.155833	0.944583	1.18 × 10^-6^
TMPO	GTF2E2	0.017083	0.934583	6.54 × 10^-10^
SMARCA4_2	RBBP4	0.005833	0.894583	7.67 × 10^-8^
CD79A	DHPS	0.011667	0.894167	1.32 × 10^-7^
FAH	MCM3	0.02375	0.877083	5.90 × 10^-7^
FAH	PSMA6	0.02875	0.790833	5.80 × 10^-6^

**Table 3:** Average confidence values for Markov relations features for the 7 highest-confidence gene pairs derived from the Golub *et al*. data [17] in a BN analysis using no prior seeds or priors derived from the literature.

*Gene pairs*		*Average no prior*	*Average prior*	*t-test p-value*
			
SMARCA4_2	SMARCA4	0.4575	0.965	0.001473
SRP9	CBX1	0.476667	0.96	0.001508
GTF2E2	TMPO	0.148333	0.938333	2.54 × 10^-6^
RBBP4	SMARCA4_2	0.15375	0.912083	1.43 × 10^-5^
CD79A	DHPS	0.135417	0.895833	1.35 × 10^-5^
FAH	MCM3	0.187917	0.881667	4.93 × 10^-5^
FAH	PSMA6	0.283333	0.79625	0.001547

The t-test p-value measures the likelihood that prior and no-prior results are equivalent.

### BN analysis of gene expression in Leukemia II: pathway reconstruction and validation

Although the analysis of the Golub *et al*. data provides some level of confidence that we can faithfully recover pair-wise interactions between genes using a seeded BN approach, we developed this approach in hopes of deducing the structure of biological networks from gene expression data. The Hu6800 GeneChip™ used in the Golub *et al*. study surveys only a limited subset of the genes within the genome and has significant gaps in nearly every pathway relevant to understanding the cellular mechanisms that distinguish ALL from AML.

Consequently, we turned our attention to the analysis of two more extensive Leukemia datasets from Ross and colleagues [18,19], who collected expression profiles on the Affymetrix U133A GeneChip™, which contains probes for more than 22,000 transcripts representing nearly the entirety of the protein-coding genes encoded within the human genome. The raw CEL files were normalized and the resulting dataset was filtered to eliminate adult low quality samples (details in Supplement S4; see Additional File 1); this left 120 pediatric ALL and 120 pediatric AML samples. To evaluate network reconstruction, we limited our analysis to genes mapping to the KEGG cell cycle pathway. For these genes, we performed a BN analysis using no prior information as well as seeds derived from the literature, PPI data, and the combination; in each case, 100 bootstrap iterations were performed using hill climbing and the BDe score. Networks were constructed using gene pairs with bootstrap confidence greater than a threshold value in the directed edge, Markov relation, and order relation features.

Given a set of genes and a predicted network describing their associations, the optimal method for validating the network would be to experimentally test each interaction that comprise the associated graph. As this is often impractical, as an alternative, we will rely the KEGG pathway database [20] as a source of "verified" interactions and network topologies that can be used to assess the fidelity of our predicted networks. For the analysis presented here, we assume that interactions represented in KEGG are true and that those not in KEGG are false. While the former is probably a good assumption, the latter is likely not; there may be as yet undiscovered interactions and, indeed, the goal of a BN analysis is to discover these. A second potential limitation of using KEGG pathways is that they are typically represented as undirected graphs while the results of a BN analysis are directed acyclic graphs. Consequently, we cannot assess the directionality of predicted edges, only their presence or absence. Further, KEGG represents physical interactions between proteins, not the RNA transcripts which are assayed in microarray studies. The seeded BN approach that we describe here models interactions between gene expression states and these may not be identical to the interaction states of the corresponding proteins. Despite these limitations, we chose to compare our results to known pathways annotated in KEGG because it is a conservative way to evaluate the performance of our approach as it does not rely on the validation of potentially novel interactions learned from the data.

**Figure 3:**
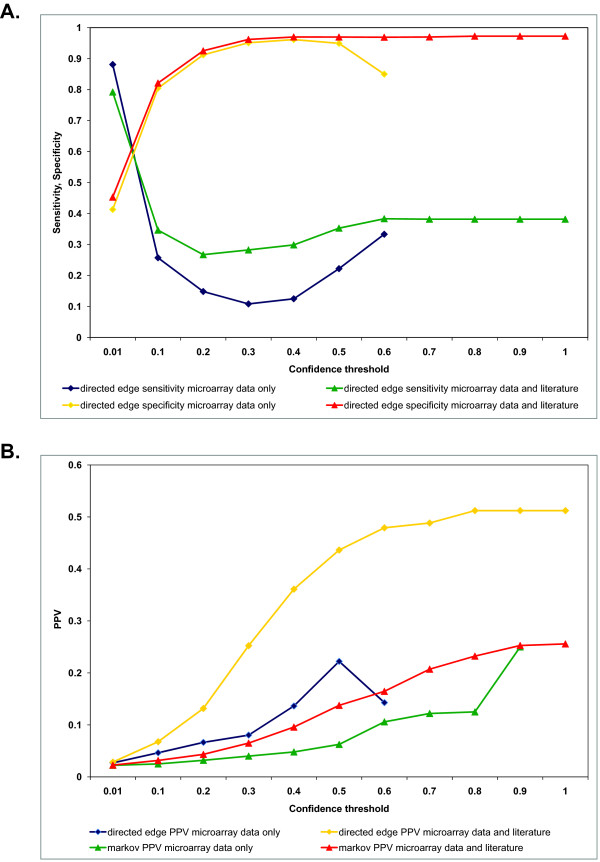
**Sensitivity, specificity tradeoff (A) and PPV (B) vs. confidence threshold when using microarray data alone or seeds derived from literature.** The Bayesian networks were learned from 100 bootstrap iterations using the hill climbing algorithm and BDe score using the leukemia datasets ofRoss *et al*. [18,19]. The learned networks were compared to corresponding subgraphs of KEGG cell cycle pathway (KEGG ID: hsa04110).

Consider an ideal graph G_I _(an undirected graph from KEGG) and a learned graph G_L _(a DAG learned using the seeded BN approach). As graphs are comprised of nodes and edges, one can compare G_I _and G_L _by comparing their sets of edges (E_I _and E_L_, respectively) and calculate true positives (TP), false positives (FP), true negatives (TN), and false negatives (FN) using a contingency table such as that shown in Table 4 and described in more detail in Supplement S5; see Additional File 1). If we define sensitivity as the probability of recovering a true edge and specificity as the probability of suppressing a false edge, then we observe a trade-off between sensitivity and specificity as a function of confidence threshold in networks constructed using microarray data only or microarray data and literature (Figure 3A). As the confidence threshold increases, sensitivity decreases and specificity increases. The positive predictive value (PPV) is the proportion of true positives among all positives (*PPV = TP/[TP+FP]*). In other words, PPV is the probability that in case an edge is recovered in the BNs, it is really a true edge as it is in KEGG. It can be seen that PPV increases with confidence threshold (Figure 3B).

**Table 4:** Contingency table for comparing learned networks to KEGG pathways.

	Learned Network		
	
Ideal (KEGG pathway)	Edge in network	Yes	No
	
	Yes	TP = |E_L_| ⋂ |E_I_|	FN = |E_I_| - TP
	No	FP = |E_L_| - TP	TN = [(|V_I_|•|V_I _- 1|)/2] - FN

Here E_L _and E_I _represent the edges in the learned and ideal graphs, respectively, where the ideal graphs are those derived from the KEGG pathways; V_I _is the set of nodes (vertices) in the ideal graph.

**Figure 4:**
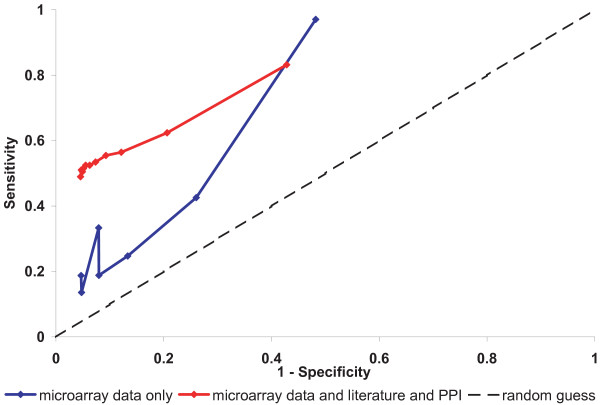
**ROC curve for Markov relations for networks deduced from the Ross *et al*.**[18,19]**data either with or without network seeds (literature plus PPI), based on 100 bootstrap iterations.** The learned networks were compared to corresponding subgraphs of KEGG cell cycle pathway (KEGG ID: hsa04110) and indicate much better overall performance for networks derived using network seeds.

A Receiver-Operator Characteristic (ROC) curve, which compares sensitivity and specificity directly (TP rate vs. FP rate [35]), suggests that the identification of Markov relations using Bayesian networks is conservative as they are found with strong evidence only at low true positive rates. Figure 4 shows ROC curves for Markov relation detection using either microarray data alone (blue) or with seeds derived from combined literature and PPI priors (red); for both, bootstrap confidence decreases as sensitivity increases. As can be clearly seen, the use of prior network seeds greatly improves our ability to detect known interactions, particularly when considering those with strong bootstrap support.

**Figure 5:**
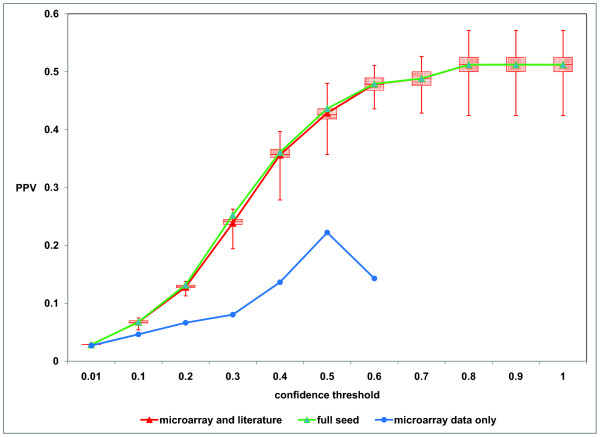
**For the Ross *et al*.**[18,19]**data, we began with our original literature network seed and systematically deleted each individual gene, learning Bayesian networks through 100 bootstrap iterations both with and without these altered literature priors.** Shown here is the positive predictive value (PPV) for identifying directed edges as a function of bootstrap confidence.

Finally, we attempted to make an estimate of our confidence in new interactions that we learn using prior network seeds. To do this, we used the Ross data [18,19] and again compared Bayesian Networks learned from the data with and without literature network priors. However, for each gene in the literature network, we systematically deleted the corresponding node, one by one, from the seed network. With this modified seed network, we performed 100 bootstrap iterations to determine how often we re-learned true directed edges (as defined by the corresponding KEGG pathway) associated with that gene relative to how often we learned the same true edges when using no prior. Figure 5 shows the average positive predictive value as a function of bootstrap confidence for edges associated with the gene deleted from the literature network, averaged over all genes. As can be seen, the use of a prior network that approximates the real network allows us to more accurately learn true interactions than is possible when not using prior information. Furthermore, BNs obtained by removing each node in the seed network yield PPVs that are very close to the PPV obtained by using the original seed network. This suggests that even when missing some information, a seed network can introduce a useful bias in the search space explored and lead to identification of high-confidence interactions.

Taken together, the evidence here, based on a number of measures, suggests that use of prior network seeds, compared to the analysis of gene expression alone, improves our ability to learn interactions known to occur between gene products and consequently to reconstruct networks in a BN framework. In addition, our analysis suggests that, ultimately, the network model that one chooses to use requires a balance between re-learning known edges and discovering new potential interactions; the choice of which to emphasize depends of the goal of the analysis. Regardless of the choice, the value of a Bayesian Network framework is that it not only provides a network graph, but conditional probabilities on the edges that result in predictive models that can be used in a variety of applications.

## Discussion

Much of the excitement generated by microarrays and other high-throughput technologies was based on the expectation that they would lead us to uncover the pathways and networks that provide the link between genotype and phenotype. Application of Bayesian Network analysis, although computationally expensive, represented one possible way to discover these important links in expression data, but failed to provide much insight when applied to the vast majority of expression datasets. Here we present a way to recover at least some of that lost promise.

In an analysis of two gene expression datasets, both representing the types of class-comparisons that are typical in microarray studies, we established that the use of domain-specific knowledge in the form of prior network seeds can improve the ability of Bayesian Network analyses to learn known interactions between genes. We demonstrated this by showing improvements in recovery of known pairwise interactions between gene products as defined by pathways in the KEGG database. Through the use of bootstrapping, we are able to assign confidence values to the individual interactions. Finally, these interactions, represented by an edge between two gene nodes, can be used to reconstruct predictive networks at any confidence level.

Generally, one would hope to use such an analysis to discover new potential interactions and to build testable models that can be validated. Not surprisingly, we find a tradeoff between sensitivity and specificity in detecting interactions when varying the bootstrap confidence threshold. Networks constructed with high confidence edges give high specificity but miss many interactions, resulting in lower sensitivity and fewer potentially novel interactions. Setting the confidence threshold too low, on the other hand, may lead to the identification of many spurious edges and limit the ultimate utility of any network model. Managing this tradeoff must, therefore, be done in the context of the experimental problem being investigated and one's ability to validate the results.

There are a number of potential limitations to what we present here. First, in the analysis of the Ross data, we chose to focus on genes within the cell cycle regulatory pathway rather than looking at the entire collection of genes in the array. However, even with this focused collection of genes, we were able to validate our hypothesis, that prior network seeds derived from the literature and PPI data improve our ability to detect known interactions relative to a standard, unseeded BN analysis. What is most exciting about the work we present here is that we were able to learn the structure of a dynamic process, the cell cycle, from a dataset consisting of static snapshots of two phenotypic states. This suggests that directed experiments where a particular network or pathway is perturbed and followed over time may further improve the overall performance of a BN approach. Using such an approach in an iterative manner, in which a network is first learned, then perturbed and the resulting data used to refine the predicted network structure, may allow us to discover novel players in many known networks and to learn previously unknown networks from DNA microarray expression profiles.

Second, it should be noted that the networks we learn from BN analysis do not represent physical networks but instead capture subtle relationships between the states of various genes and their ability to influence the states of others. Nevertheless, in our evaluation of BN performance, we compared our results to physical interaction networks between proteins represented in the KEGG database. We believe this is justified as the hypothesis underlying our work is that the physical relationship captured in gene-product interactions is reflected in transcriptional response and the results we present, showing good correlation between the physical and BN networks provide some validation for this hypothesis.

Finally, one may consider whether the use of seeds biases us toward simply re-learning known networks documented in the literature. While we believe the evidence presented in this manuscript suggests otherwise, even if it were the case, our approach represents an automated way of extracting network graphs from a gene list, refining the graph through the use of expression data, and learning conditional probabilities that can be used to make predictions as to how the system may respond to perturbations. The implications of this for a wide range of problems ranging from mechanistic studies to drug target prioritization remain to be explored.

## Conclusion

Ultimately, the value of what we present here will be judged based on its utility. The goal of most systems biology research is the development of predictive, testable models that can be used to infer the properties of biological systems. The seeded BN approach we describe here yields such models and the data we present indicates that the networks we learn do, indeed, reflect the properties of biological systems. While we await further validation of the approach, we believe that seeded Bayesian Networks will be an important new approach for the analysis of genome-scale expression data.

## Abbreviations

ALL: Acute Lymphoblastic Leukemia; AML: Acute Myeloid Leukemia; BNs: Bayesian Networks; CPDAG: complete partially directed acyclic graph; DAG: directed acyclic graph; DFS: depth-first search; FN: false negatives; FP: false positives; KEGG: Kyoto Encyclopedia of Genes and Genomes; PPI: protein-protein interaction; PPV: positive predictive value; ROC: Receiver-Operator Characteristic; TP: true positives; TN: true negatives.

## Authors' contributions

JQ and AD conceived of, developed, tested, validated the method presented here, and wrote this manuscript. AD performed the initial software implementation; JQ and his group adapted it for use in the MeV software tool [36-38]

## Supplementary Material

Additional file 1 Supplemental Methods and Results. This additional file contains five supplements: Supplement S1: Directing Seed Networks, Supplement S2: Equivalence classes of DAGs, Supplement S3. PPI-extended "top 40" gene list from Golub et al [17], Supplement S4: Preprocessing of the Ross et al. ALL/AML leukemia datasets [18,19], Supplement S5: Model for estimating performance. Click here for file
