# Supplementary material for: Seeded Bayesian Networks: Constructing genetic networks from microarray data
Source: BMC Syst Biol. 2008 Jul 4;2:57. doi: 10.1186/1752-0509-2-57 (PMC2474592; doi:10.1186/1752-0509-2-57)
Supplement: Additional file 1 — Supplemental Methods and Results. This additional file contains five supplements: Supplement S1: Directing Seed Networks, Supplement S2: Equivalence classes of DAGs, Supplement S3. PPI-extended "top 40" gene list from Golub et al [17], Supplement S4: Preprocessing of the Ross et al. ALL/AML leukemia datasets [18,19], Supplement S5: Model for estimating performance. [file 1752-0509-2-57-S1.doc]

**Supplemental Materials:**

**Supplement S1: Directing Seed Networks**

The methods we use to derive networks based on the literature or protein-protein interactions are undirected and merely show associations between genes. However, as a seed for a BN search, the starting network should be a directed acyclic graph (DAG). So the problem we face is that, given an undirected graph, G, we need to produce a DAG with as many edges from G as possible. Here we describe two methods. The first is a heuristic approach based on gene ontology (GO) terms and the second, and the one we use in the discussion presented in our manuscript, is a modification of the Depth-First Search Algorithm.

(1) Using GO terms:

Algorithm direct using GO terms

Input: undirected G(V,E) Output: DAG G’(V’,E’)

V’ = V

E’ = empty set

for each e = (u,v) in E

if(|E’| == |V| -1) return G’(V’,E’)

else

if(GO (u)) == TF && (GO(v) != TF)

add (u->v) to E’

if(GO (u)) != TF && (GO(v) == TF)

add (v->u) to E’

else // either both or neither of u,v are TF

flip a coin to decide (u,v) direction

end if

end if

end for

This approach produces a tree with directed edges which, by definition does not contain cycles and is therefore a DAG.

(2) Using Modified Depth-First Search (DFS):

Algorithm direct using modification of depth-first search

Input: undirected G(V,E) Output: DAG G’(V’,E’)

G’ = empty graph

Run Depth-first search on V

Add resulting depth-first tree to G’(V’,E’)

for each back edge e = (u,v) found in E

if visiting timestamp(u) < visting timestamp(v)

add (u -> v) to E’

else

add (v -> u) to E’

end if

end for

Depth-first search is commonly used for cycle detection (1) and our modification builds on this application by reversing edges that explicitly create cycles. The algorithm we use first runs a standard depth-first search on an given undirected graph (see (1) for a discussion of DFS and pseudocode; a description and pseudocode can also be found at http://en.wikipedia.org/wiki/Depth-first_search). Then starting with the resulting depth-first tree, adds edges in the reverse order of that which indicates a directed cycle: back edges in increasing order of visiting timestamp. This procedure will result in a DAG as it will not introduce any new cycle. A proof of this follows immediately.

Claim:

Adding reverse edges than back edges that lead to cycle detection to a tree does not create a new cycle.

Proof by contradiction:

Suppose this claim is false, *i.e.* suppose adding a back edge in increasing order of visiting timestamp to a tree does create a new cycle. Without loss of generality, let *v* be visited before *u* during the depth-first search. Let *(u -> v)* be the back edge in decreasing order of visiting timestamp which would have created a cycle. Let *u,v, v_1_, …, v_m_, u* be this cycle.

We will add this back edge in increasing order of visiting timestamp *(v -> u)*.

Now adding *(v -> u)* will create a new cycle: *v, u, u_1_, …, u_n_,v*. Then *u, u_1_, …, u_n_, v, v_1_, …, v_m_, u* is a cycle not containing *(v->u)*. But the starting graph was a tree which by definition does not contain any cycles. This is a clear contradiction.

Therefore, adding *(v->u)* reverse edge than back edge which leads to cycle detection does not create any new cycle as claimed.

**Supplement S2: Equivalence classes of DAGs**

After learning BNs, it may be statistically impossible to distinguish one resulting DAG from another DAG. For this reason, we need to consider equivalence classes of DAGs: two graphs are equivalent if they both imply the same set of independencies.

Graphs belong to an equivalence class if and only if they have the same underlying undirected graph and v-structures (directed edges converging to the same node) (2). A partially directed acyclic graph (PDAG) is a graph where some edges are directed and some are undirected and one cannot trace a cycle by following the direction of directed edges and any direction for undirected edges. A PDAG can represent an equivalence class of graphs such that if a PDAG contains a directed edge X → Y, it implies that all members of the equivalence class contain the directed edge X → Y, and if a PDAG contains an undirected edge (X, Y) then some members contain X → Y and others contain Y → X. Compelled edges are edges that must be directed the same way in all DAGs belonging to the equivalence class of the given DAG. Reversible edges are edges that may be directed differently in at least one DAG belonging to the equivalence class of the given DAG as compared to the given DAG. A complete PDAG (CPDAG) is a PDAG for which each compelled edge in the equivalence class is directed and each reversible edge in the equivalence class is undirected. A DAG to CPDAG algorithm takes as input a DAG and outputs a CPDAG representation of the equivalence class to which this DAG belongs (3).

**Supplement S3. PPI-extended “top 40” gene list from Golub et al.**

As noted in the text, we began with data from Golub and colleagues (4) and identified the 40 genes that were most significant for distinguishing ALL from AML. This initial set was extended using the protein-protein interaction data to include those genes a distance three of fewer from the initial set (based on PPI interactions) resulting in a set of 63 genes; the resulting literature plus PPI “seed” network, included 44 gene nodes connected by 48 edges and 19 singleton (unconnected) genes. The PPI-extended gene list is included below.

| GB Accession | Affymetrix Hh6800 ID | Gene Symbol | Annotation |
| --- | --- | --- | --- |
| AF009426 | AF009426_at | C18orf1 | chromosome 18 open reading frame 1 |
| D13633 | D13633_at | DLG7 | discs, large homolog 7 (Drosophila) |
| D31767 | D31767_at | DAZAP2 | DAZ associated protein 2 |
| D63874 | D63874_at |  |  |
| D63880 | D63880_at | CNAP1 | chromosome condensation-related SMC-associated protein 1 |
| D80004 | D80004_at | KIAA0182 | KIAA0182 protein |
| D83032 | D83032_at | ZNF638 | zinc finger protein 638 |
| D84110 | D84110_at | RBPMS | RNA binding protein with multiple splicing |
| D87458 | D87458_at | TRIM9 | tripartite motif-containing 9 |
| D88378 | D88378_at | PSMF1 | proteasome (prosome, macropain) inhibitor subunit 1 (PI31) |
| HG2573-HT2669 | HG2573-HT2669_at |  |  |
| HG511-HT511 | HG511-HT511_at |  |  |
| J05243 | J05243_at | SPTAN1 | spectrin, alpha, non-erythrocytic 1 (alpha-fodrin) |
| L13278 | L13278_at | CRYZ | crystallin, zeta (quinone reductase) |
| L36844 | L36844_at | CDKN2B | cyclin-dependent kinase inhibitor 2B (p15, inhibits CDK4) |
| L47738 | L47738_at | CYFIP2 | cytoplasmic FMR1 interacting protein 2 |
| M15796 | M15796_at | PCNA | proliferating cell nuclear antigen |
| M33308 | M33308_at |  |  |
| M55150 | M55150_at | FAH | fumarylacetoacetate hydrolase (fumarylacetoacetase) |
| M81933 | M81933_at | CDC25A | cell division cycle 25A |
| M92287 | M92287_at | CCND3 | cyclin D3 |
| S50223 | S50223_at | ZNF22 | zinc finger protein 22 (KOX 15) |
| U00952 | U00952_at | PBXIP1 | pre-B-cell leukemia transcription factor interacting protein 1 |
| U09579 | U09579_at | CDKN1A | cyclin-dependent kinase inhibitor 1A (p21, Cip1) |
| U09953 | U09953_at | RPL9 | ribosomal protein L9 |
| U20998 | U20998_at | SRP9 | signal recognition particle 9kDa |
| U22897 | U22897_at | NDP52 | nuclear domain 10 protein |
| U31501 | U31501_at | FXR2 | fragile X mental retardation, autosomal homolog 2 |
| U32944 | U32944_at | DNCL1 | dynein, cytoplasmic, light polypeptide 1 |
| U35451 | U35451_at | CBX1 | chromobox homolog 1 (HP1 beta homolog Drosophila ) |
| U40343 | U40343_at | CDKN2D | cyclin-dependent kinase inhibitor 2D (p19, inhibits CDK4) |
| U59321 | U59321_at | DDX17 | DEAD (Asp-Glu-Ala-Asp) box polypeptide 17 |
| U63825 | U63825_at | DIPA | hepatitis delta antigen-interacting protein A |
| U78798 | U78798_at | TRAF6 | TNF receptor-associated factor 6 |
| U79262 | U79262_at | DHPS | deoxyhypusine synthase |
| U79304 | U79304_at | FAM13C1 | family with sequence similarity 13, member C1 |
| U82759 | U82759_at | HOXA9 | homeo box A9 |
| U90909 | U90909_at | C14orf32 | chromosome 14 open reading frame 32 |
| U96113 | U96113_at | WWP1 | WW domain containing E3 ubiquitin protein ligase 1 |
| U96114 | U96114_at | WWP2 | WW domain containing E3 ubiquitin protein ligase 2 |
| X13956 | X13956_at | MGC10471 | hypothetical protein MGC10471 |
| X16282 | X16282_at | ZNF250 | zinc finger protein 250 |
| X52142 | X52142_at | CTPS | CTP synthase |
| X59417 | X59417_at | PSMA6 | proteasome (prosome, macropain) subunit, alpha type, 6 |
| X63469 | X63469_at | GTF2E2 | general transcription factor IIE, polypeptide 2, beta 34kDa |
| X64044 | X64044_at | U2AF2 | U2 (RNU2) small nuclear RNA auxiliary factor 2 |
| X67337 | X67337_at | CPSF6 | cleavage and polyadenylation specific factor 6, 68kDa |
| X69838 | X69838_at | BAT8 | HLA-B associated transcript 8 |
| X74262 | X74262_at |  |  |
| X76648 | X76648_at | GLRX | glutaredoxin (thioltransferase) |
| X84373 | X84373_at | NRIP1 | nuclear receptor interacting protein 1 |
| Z19002 | Z19002_at | ZBTB16 | zinc finger and BTB domain containing 16 |
| Z50194 | Z50194_at | PHLDA1 | pleckstrin homology-like domain, family A, member 1 |
| M61853 | M61853_at | CYP2C18 | cytochrome P450, family 2, subfamily C, polypeptide 18 |
| Z15115 | Z15115_at | TOP2B | topoisomerase (DNA) II beta 180kDa |
| AF012024 | AF012024_s_at | ITGB1BP1 | integrin beta 1 binding protein 1 |
| X62153 | X62153_s_at | MCM3 | MCM3 minichromosome maintenance deficient 3 (S. cerevisiae) |
| Y08766 | Y08766_s_at | SF1 | splicing factor 1 |
| M31211 | M31211_s_at | MLC1SA | myosin light chain 1 slow a |
| M65214 | M65214_s_at | TCF3 | transcription factor 3 (E2A immunoglobulin enhancer binding factors E12/E47) |
| U09087 | U09087_s_at | TMPO | thymopoietin |
| X95632 | X95632_s_at | ABI2 | abl interactor 2 |
| U29175 | U29175_at | SMARCA4 | SWI/SNF related, matrix associated, actin dependent regulator of chromatin, subfamily a, member 4 |

**Supplement S4:** **Preprocessing of the Ross et al. ALL/AML leukemia datasets**

We assembled microarray data from published studies by Ross and colleagues. The first (5) profiled 132 ALL samples on the Affymetrix U133A and B GeneChips™; the second (6) analyzed expression in 130 pediatric and 20 adult AML samples on the Affymetrix U133A array; all data are available at the following URLs (but have not been submitted to GEO or ArrayExpress):

1. http://www.stjuderesearch.org/data/ALL3
2. http://www.stjuderesearch.org/data/AML1

*Preprocessing the samples*

First, we limited our analysis to pediatric cases only and to those data collected on the U133A array. This included all of the ALL data from the U133A arrays (5) but eliminated the 20 adult AML samples leaving 130 pediatric AML expression profiles (6). Raw CEL files were downloaded from the St. Jude’s website and preprocessed with MAS5 using default parameters to obtain initial quality control data: percent present, scaling factor, gapdh and actin ratios. All CEL files were then normalized using RMA with background correction and quantile normalization and then globally scaled such that the median intensity for each chip is the same across all samples. Using this normalized, scaled data, we eliminate outliers in either the detection percentage (μ*_d_* – 2σ*_d_*) or in the scaling factor (μ*_s_* + 2σ*_s_*) as these typically represent data from poor quality RNA samples that produce low-intensities on the arrays; this eliminated 22 samples.

*Preprocessing the genes*

From the 22,283 genes represented on the Affymetrix U133A array, we eliminate those without any present calls in either sample class. Among the ALL samples 6,173 genes lacked present calls and in the AML samples there were 5,431 genes without present calls; the union of these is a set of 6,895 genes and these were eliminated from further analysis.

**Supplement S5: Model for estimating performance**

Estimating the performance of the seeded BN approach requires that we define our ability to recover real edges represented in the KEGG pathways we use as “ground truth” relative to how often we learn edges that are not represented in those pathways.

Let the total number of positives be equal to the number of edges in the KEGG pathway, P = |E _I_|; in comparison to the learned graph from our BN analysis, this can be represented as the sum of true positives (TP) and false positives (FP), P = TP + FP. Here we can represent TP as the number of edges in common between the learned (G_L_) and ideal (G_I_) graphs, TP = |E_L_| ∩ |E_I_|. We define the number of False Positives in our BN as the difference between the learned edges and the true positives, FP = |E_L_| - TP, *i.e.*, the number of edges extra in E _R_ that are not in E_I_. The number of False Negatives is the number of edges missing from the learned graph, FN = |E_I_| - (|E_L_| ∩ |E_I_|). Finally, the number of True Negatives is the difference between the total number of possible edges, [(|V_I_|•|V_I_ -1|)/2], and the number of false negatives, TN = [(|V_I_|•|V_I_ - 1|)/2] – FN.

The total number of negatives, N = |V _I_|.|V _I_ -1|/2, corresponds to the number of possible edges in the graph. The total number of negatives is the sum of true negatives (TN) and false negatives (FN). Let FN = |E_I_| - (|E_L_| ∩ |E_I_|) which corresponds to the number of edges from G _I_ which are missing in G _R_. Let TN = N – FN. In other words, the number of true negatives is the number of possible edges minus the number of missing edges.

**References to the Supplement**

1. Cormen, T.H., Leiserson, C.E. and Rivest, R.L. (1990) *Introduction to Algorithms*. MIT Press, Cambridge, Massachusetts.

2. Verma, T. and Pearl, J. (1990), *Proceedings of the Sixth Conference on Uncertainty in Artificial Intelligence*, pp. 220-227.

3. Chickering, D.M. (1996) In Horvitz, E. and Jensen, F. (eds.), *Proceedings of the Twelfth Conference on Uncertainty in Artificial Intelligence*. Morgan Kaufmann, pp. 150-157.

4. Golub, T.R., Slonim, D.K., Tamayo, P., Huard, C., Gaasenbeek, M., Mesirov, J.P., Coller, H., Loh, M.L., Downing, J.R., Caligiuri, M.A. *et al.* (1999) Molecular classification of cancer: class discovery and class prediction by gene expression monitoring. *Science*, **286**, 531-537.

5. Ross, M.E., Zhou, X., Song, G., Shurtleff, S.A., Girtman, K., Williams, W.K., Liu, H.C., Mahfouz, R., Raimondi, S.C., Lenny, N. *et al.* (2003) Classification of pediatric acute lymphoblastic leukemia by gene expression profiling. *Blood*, **102**, 2951-2959.

6. Ross, M.E., Mahfouz, R., Onciu, M., Liu, H.C., Zhou, X., Song, G., Shurtleff, S.A., Pounds, S., Cheng, C., Ma, J. *et al.* (2004) Gene expression profiling of pediatric acute myelogenous leukemia. *Blood*, **104**, 3679-3687.
